# Computational Methodology for Absolute Calibration Curves for Microfluidic Optical Analyses

**DOI:** 10.3390/s100706730

**Published:** 2010-07-12

**Authors:** Chia-Pin Chang, David J. Nagel, Mona E. Zaghloul

**Affiliations:** Department of Electrical and Computer Engineering, The George Washington University, Washington DC 20052, USA; E-Mails: nagel@gwu.edu (D.J.N.); zaghloul@gwu.edu (M.E.Z.)

**Keywords:** microfluidic, chemical analysis, bio-chemical analysis, optical fluorescence, optical absorption

## Abstract

Optical fluorescence and absorption are two of the primary techniques used for analytical microfluidics. We provide a thorough yet tractable method for computing the performance of diverse optical micro-analytical systems. Sample sizes range from nano- to many micro-liters and concentrations from nano- to milli-molar. Equations are provided to trace quantitatively the flow of the fundamental entities, namely photons and electrons, and the conversion of energy from the source, through optical components, samples and spectral-selective components, to the detectors and beyond. The equations permit facile computations of calibration curves that relate the concentrations or numbers of molecules measured to the absolute signals from the system. This methodology provides the basis for both detailed understanding and improved design of microfluidic optical analytical systems. It saves prototype turn-around time, and is much simpler and faster to use than ray tracing programs. Over two thousand spreadsheet computations were performed during this study. We found that some design variations produce higher signal levels and, for constant noise levels, lower minimum detection limits. Improvements of more than a factor of 1,000 were realized.

## 1. Introduction

The qualitative and quantitative chemical and bio-chemical analyses of micro-liter and smaller volumes of diverse fluids constitute one of the main applications of microfluidic systems [1]. There are several approaches to obtaining signals from micrometer-scale volumes in the process of performing analyses [2–6]. Electrical measurements are common for samples that have an ionic component (DC conductivity) or polarizable molecules (AC impedance). Optical techniques, notably fluorescence and also absorption, are also widely used for samples that are optically active [7,8].

As part of an experimental study on the limits of detection for analyte molecules in micro-channels or thin films, we are concerned with relating the absolute number of molecules accessible to optical emission and absorption equipment to the absolute signal strengths (usually in volts) that are available from analytical instruments. This paper provides the set of linked equations for such relationships for both optical emission and absorption measurements. There is considerable literature on chemical and biological analytical calibration curves for microfluidic systems, but most calibration curves are not on an absolute basis. Further, no papers provide a complete description of the components and geometries employed. In this paper, we present and use a new and straightforward computational approach for quantitative optical analysis of microscale fluids. Absolute calibration curves were calculated for 216 varying designs and concentrations.

There are several advantages to the technique we have developed for optical micro-analyses of fluids. Most fundamentally, it deals with individual entities. These are the molecules, which are the object of using microfluidic analytical systems, and quanta, specifically photons and electrons, that are employed for the analyses. Our approach focuses on the individual components in an optical micro-analytical system, each with associated specifications, efficiency and geometry, which determine the overall performance of the system. We present simple and useful equations that link the components optically. They determine the transport of photons through the system. Overall, use of the equations relates the number of molecules in the analytical volume to the measured signal. This approach makes it relatively easy to determine the components or geometries that are most amenable to significant improvements during design of an analyzer system. In fact, the variation of the measured signal with changes in any of the component parameters is straightforward to compute, if the geometrical and other parameters are known or estimated. Calculations based on the method can be made using simple computer programs or even spreadsheets.

This paper provides three benefits. First, we developed and utilized a comprehensive, yet efficient, means of computing absolute calibration curves for microfluidic optical analysis systems. Second, the numerous results reported and discussed clearly demonstrate the advantages of this methodology for examining the efficacy of alternative optical components and designs. Finally, we have a computational basis for comparison with experiments.

More specifically, the main features of our new methodology can be summarized as follows:

- It is absolute, and relates molecular concentrations or numbers to realistic detector signals.
- It is complete, including all components and geometrical factors that affect the measured signal for a given analyte concentration.
- The methodology is almost entirely algebraic, except for the case of fiber optic coupling to microchannels, which is not very important practically.
- The effects of the various parameters needed for computations are quite clear.
- Being mathematically simple, the method makes possible fast calculations and thorough parametric studies.
- The technique permits examination of realistic designs without the time and expense of making and using prototypes.
- The calculation of calibration curves is much more efficient than to measuring them in the laboratory.
- The methodology is testable by comparisons of its predictions with the results of experiments using the same components and geometries.
- The methodology is scale-independent. It can be used for macroscopic, mesoscopic and microscopic optical systems.

**Figure 1.**
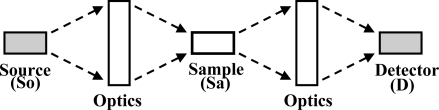
The sequence of major components in an optical micro-analytical system. For emission measurements, the source light goes as far as the sample, where the new fluorescent light originates. For absorption measurements, the two sets of optics and the sample can be thought of as the entire optical system coupling the source to the detector.

Our interest in emission and absorption methods of optical micro-analysis has another basis, namely their similarity. This is indicated schematically in Figure 1. In both cases, a source of light is needed. In the emission case, the light is absorbed, and that stimulates fluorescence from molecules in the sample or from tags attached to them. In the absorption case, the source provides the photons that probe the sample and are fractionally absorbed within it. Both emission and absorption methods usually involve a variety of optics between the source and sample in order to collect light from the source and focus it on the sample. Similarly, optics are commonly used between the sample and detector to collect emission or unabsorbed photons from the sample and focus them on a detector. Optics in both positions may give spectral discrimination to provide molecular specificity or give other benefits, notably background reduction. The quantitative transport of photons from the source to the sample to the measuring equipment depends on the optical efficiency of the individual components, and many geometrical and spectral factors.

The next section presents our computational methodology for quantitative analysis of samples in micro-channels or thin films by absorption or fluorescence. Section 3 provides many illustrative computed calibration curves, which were obtained using the methodology. These results are discussed in the following section. The last section sketches what is needed for future experimental work on quantitative microfluidic optical analyses.

## 2. Computational Methodology

We seek to compute the output of the detector in a microfluidic optical analytical system as a function of the concentration or the number of molecules accessible to the system. Such a relationship constitutes the useable part of the calibration curve for the instrument. That is the part of a calibration curve above the noise level of the signal and below the saturation of the system output. The computation requires linking the source of photons for stimulating fluorescence or probing absorption to the analytical sample and detector through all intermediate optics and spectrally sensitive components. Geometry plays a dominant role in the efficiency with which all the components are coupled. In this section, we provide equations and diagrams for the needed calculations. Concatenation of all the equations for a particular set of components and their arrangement yields the desired calibration curve. We emphasize that we are sacrificing some detail for completeness. We provide relatively simple, but useful equations for a complete linkage. Uncertainties in our results are small compared to the large variations in optical design, which can change calibration curves by more than three orders of magnitudes for the same concentration of the analyte.

The quantitative presentation of our methodology is for both fluorescence and absorption measurements of samples in both microchannels and thin films with lens, no optics or fiber optic coupling of the source to the sample and the sample to the detector. The light from the source will be assumed to strike the samples in the channels or films normally, with one exception. That is coaxial fiber coupling into and out of the ends of microchannels. It is relatively difficult and unproductive to use lenses to couple light from a source into the axis of a microchannel. After considering the primary aspects of lens coupling, we will turn to the possibility of dispensing with geometrical optics entirely. Then, we consider the use of fiber optics. Fibers also make it possible to do either fluorescence or absorption measurements along the length or perpendicular to the axis of a microchannel. The use of optical fibers with thin film samples is usually not reasonable because either very little of the sample film is viewed or the instrument becomes relatively large. However, our methodology can be applied to that case also.

The following paragraphs trace the source or fluorescent photons from component to component, for samples in microchannels or thin films. It is assumed throughout that the components of the system are properly aligned. Achieving alignment is challenging but must be done experimentally, if performance is to be optimized, or if comparisons of computed and measured signals are to be made.

Again, we emphasize that, for absorption measurements, the S_o_ to S_a_ and S_a_ to D axes on both sides of the samples must be co-linear. However, that is neither necessary nor desirable for fluorescence measurements because light from the source that transits the sample might strike and stimulate the detector as a very undesirable background. We will not explicitly treat the very diverse geometries for fluorescence measurements in which the S_o_ to S_a_ and S_a_ to D axes on the opposite or same side of the samples are not co-linear. Doing so for a specific system design (components and geometries) is straightforward.

### 2.1. Source Strength

The specifications for the intensity of LEDs are commonly given in the photometric units of lumens. Equation (1) can be used to convert lumens to watts.

(1) $$\text{Power}\;(W)=\frac{\text{Lumens}}{683\;\text{lumens}\;\text{per}\;\text{watt}\times (\text{Luminous}\;\text{Efficacy})}$$

where luminous efficacy is wavelength-dependent [9].

Laser specifications are generally given in watts. Equation (2) is used for computing the photons per second from the watts.

(2) $$P_{s}\;=\;\frac{\text{Photons}}{\text{Second}}\;=\;5.03\;\times \;10^{15}\times \;\text{Power}(W)\times \lambda (\text{nm})$$

where λ is the wavelength of the laser light.

Some light source specifications give the full conical emission beam angle (2θ). The corresponding solid angle (Ω_S_) in units of steradians is given by Equation (3):

(3) $$\Omega_{s}\;=\;2\pi (1-\text{cos}\theta )$$

### 2.2. Source to Lens to Sample

**Figure 2.**
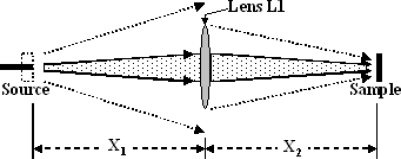
Schematic of a larger source like an LED (dotted box) or a small source such as a laser (black line), and a lens that collects the light and focuses it on the sample.

A diagram useful for computing the fraction of the photons emitted by the initial source that gets to the plane of the microfluidic sample containing the analyte is given in Figure 2. There are two primary cases. In the first, some of the source photons miss the lens and are wasted. Then, Equation (4) permits computation of the fraction of the photons that strikes the lens and gets focused onto the sample plane. Otherwise all of the photons hit the lens. The small loss of photons due to the lens itself is ignored.

(4) $$P(\mathrm{So}\;\to \;L1)\;=\;P_{S}\times \frac{\pi R_{L1}^{2}}{\Omega_{S}X_{1}^{2}}$$

P_S_ is number of photons per second the light source generates, R_L1_ is the radius of Lens L1, X_1_ is the distance between light source and Lens L1, and Ω*_s_* is the source emission solid angle in steradians.

### 2.3. Focal Conditions on the Micro-channel

**Figure 3.**
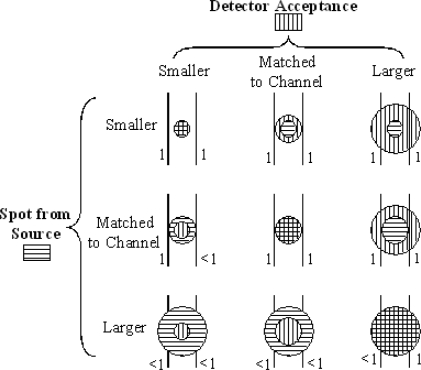
Schematic showing the relative sizes of the source focal spot, the detector acceptance region and the micro-channel, which is indicated by the two parallel vertical lines. The numbers to the left of each of the nine sketches indicate what fraction of the source photons can make it into the analyte fluid in the channel. The numbers to the right of the sketches indicate the fraction of the illuminated area at the channel, which be seen by the detector.

There are three possibilities for the relative size of the focal spot on the plane of the channel and the size of the channel. Similarly, there are three cases for the view of the detector backwards to that plane. The nine combinations are indicated in Figure 3. The essential factors are (a) the size of the source focal spot at the sample and (b) the sample area from which photons can get to the detector, both relative to (c) the width of the channel and each other. The focal spot for the source and the region viewed by the detector or spectrometer are commonly circles, although they may have rectangular or other shapes.

The area of the focused source spot on the plane of the micro-channel A_C_ can be computed from the source area A_S_, the lens focal length F_1_ and the distances between components shown in Figure 3. Equations (5) and (6) apply for a thin lens.

(5) $$A_{C}\;=\;A_{S}\;\times \frac{X_{2}}{X_{1}}$$

(6) $$\frac{1}{F_{1}}\;=\;\frac{1}{X_{1}}+\frac{1}{X_{2}}$$

X_1_ and X_2_ are both >F_1_. If they are equal and equal to 2F_1_, the area of the spot on the channel is the same as the source size. Then, ignoring the small losses in the lens, the area photon density is the same at the source and channel, when the lens intercepts all of the emitted photons.

As already noted for both emission and absorption, the collinear directions of S_o_ to S_a_ and S_a_ to D can be either (a) normal to a channel or a thin film sample, or (b) parallel to and within a channel. The first is best with lens coupling with either one-dimensional (channel) or two-dimensional (film) samples, and it will be treated next. Then, the second, which is best for fiber optic coupling, will be considered near the end of this section. Other geometries are possible, but those two limiting cases are generally most advantageous. The primary exception is to have the S_o_ to S_a_ and the S_a_ to D axes at some angle to each other in order to prevent source photons from directly entering the detector during fluorescence measurements.

### 2.4. Transmitted Light Perpendicular to a Channel or Film

For absorption, the incident and transmitted radiation can be normal to the channel or film. In that case, the number of transmitted photons P_T_ is given be Equation (7), again for the optically thin case.

(7) $$P_{T}\;=\;P_{c}e^{-\varepsilon C\ell }$$

where P_c_ is number of photons striking the fluid in the channel or film. ε is the molar absorption coefficient [10] with units of L·mole^−1^·cm^−1^ when *l* is the sample thickness, in centimeters, in the direction on a line to the source. C is the volumetric concentration (molarity) of the solution.

### 2.5. Fluorescence Perpendicular to a Channel or Film

The number of emitted fluorescent photons is equal to the number of absorbed photons times the quantum yield. Equations (8), (9) and (10) apply.

(8) $$P_{\text{abs}}\;=\;P_{c}\;(1-e^{-\varepsilon C\ell })$$

For the common case that the sample fluid is optically thin, that is, εC*l* is small compared to unity,

(9) $$P_{\text{abs}}\;=\;P_{c}\times \varepsilon \times C\times \ell$$

and

(10) $$P_{\text{Sa}}\;=\;P_{\text{abs}}\times \text{QY}$$

where P_Sa_ is number of photons sample emitted and QY is the quantum yield.

### 2.6. Sample to Lens to Filter and Detector

**Figure 4.**
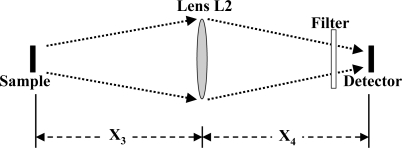
Schematic of the path for radiation from the sample, either fluorescence or transmission, through a lens and spectral filter to the detector.

The sample is effectively a source of radiation with an emission solid angle of 4π steradians for the rest of the system, when the light from the sample is fluorescence. As was the case with the source, it is necessary to compute the fraction of the photons from the sample that are intercepted by the second lens. This is illustrated in Figure 4.

A relation similar to Equation (4) is employed to compute the fraction of the fluorescent radiation from the sample that strikes the second lens L2. It is given in Equation (11).

(11) $$P(\text{Sa}\to L2)\;=P_{\text{Sa}}\;\times \;\frac{R_{L2}^{2}}{4X_{3}^{2}}$$

where R_L2_ is the radius of Lens L2 and X_3_ is the distance between sample and Lens L2. We note that, if a large area detector can be placed close to the sample, the lens L2 is not needed. However, for fluorescence measurements, this will lead to the detector intercepting and responding to unabsorbed source photons. Most of that unwanted radiation can be intercepted and absorbed by a narrow bandwidth filter in front of the detector.

For absorption computations, the angle at which transmitted radiation emerges from the analyte fluid can be determined by either (a) its entrance angle, when absorption is measured across a microchannel, or (b) the confines of the micro-channel, when absorption is measured along the length of a channel. That is, the ratio of the channel width to the length over which incident radiation propagates within the channel can determine the emergence angle.

### 2.7. Spectral Discrimination

Although a spectrometer is the best spectral discrimination tool, it will not be quantitatively considered in this methodology. In order to compute the output of a spectrometer on an absolute basis, both the wavelength-dependent input and overall spectrometer efficiency must be known. The latter is rarely available.

A useful filter is usually a narrow band interference device with peak wavelength very close to the peak wavelength of the fluorescence spectrum. The transmission characteristics of well-designed and manufactured filters permit 50% to nearly 100% transmission within a pass band that includes some or all of the entire width of the fluorescence lines, or the transmitted radiation for the absorption case. Transmitted fluorescence photons after filter can be computed as:

(12) $$P_{\mathrm{after filter}}\;=\;P(\text{Sa}\to \;L2)\times \frac{\text{FWHM of filter}\times \text{Transmission Efficiency}}{\text{Bandwidth of Emission Spectrum}}$$

A quantitative determination of the filter pass band and the fluorescent line width can be done by auxiliary measurements with a spectrometer, if they are not available from the manufacturer. Doing so will determine if any correction has to be applied in the computation of the number of photons reaching the detector [8].

### 2.8. Detector Signals

In some cases, the active area of a detector is smaller than the exposed area in the detector plane, which is irradiated by fluorescent photons. Equation (13) gives the number of photons striking the detector, namely P_D_:

(13) $$P_{D}\;=\;P_{\mathrm{after filter}}\times \frac{\text{Active area of Detector}}{\text{Exposed Area in Detection Plane}}$$

The electronic signals from the detector depend on the number of photons incident on it, the wavelength-dependent efficiency and the electronic gain, if any. Equation (14) applies.

(14) $$E_{D}\;=\;P_{D}\times \text{QE}\times G$$

where E_D_ is the number of electrons per second from the detector, P_D_ is the number of photons received by detector per second, QE is the quantum efficiency of detector, and G is the gain of the detector.

For almost all detectors, the efficiency for conversion of photons to electrons is less than unity. Quantum efficiencies are usually available from the detector manufacturer. Many detectors do not cause multiplication of the number of electrons that result from photon absorption in the detector. That is, they have no gain. However, avalanche photo diodes, and either solid-state or vacuum photomultipliers, do provide gain. The value of the gain can be high, with as many as one million electrons emerging from the detector for every electron initially generated by photo absorption. However, detectors that provide very high gains involve high voltages, to which the gain is very sensitive. Also, they are relatively expensive and, in the case of vacuum tubes, are significantly larger than solid-state detectors without gain. The latter are commonly PN or PIN diodes, which are relatively small and cheap, and require only low voltages. However, they do not have gain within the detector element. Solid state photomultipliers employ intermediate voltages and still offer substaintial gains.

Photo sensitivity (also known as responsivity) is commonly expressed as amps (coulombs per second) per watt (joules per second) of the incident light. Hence the definition of a Coulomb and Equation (2) must be employed for conversion of units. The responsivity converts the photons received by detector per second into the signal output of the detector without the use of Equation (14). If responsivity information is provided, then output signal of the detector is:

(15) $$\text{Output Signal}=\frac{P_{D}}{5.03\times 10^{15}\times \lambda }\times (\text{responsivity at Gain}=1)\times G$$

### 2.9. Post-Detection Electronics

The signals directly from individual detectors or arrays of detectors are commonly quite small and they may contain noise that is often amenable to electronic filtering. In general, signals from photon detectors are handled in either of two modes, pulse counting or current measurements. In the first case, pulses due to absorption of individual photons in a detector, usually with gain, can be counted. Then, there are some beneficial possibilities to reject noise. Electronic filters can be used to discriminate against noise with frequencies that are either too low, or else too high relative to the photon arrival and electron production rates. Electronics, which determine the height or integral of the pulses, are commonly use to reject pulses that are too small. Such electronics can perform analyses of the shape of the pulses to insure that, even if the pulses pass the size screening, they have the proper characteristics to be caused by photons. However, the very fast electronics for capture and examination of individual pulses in real time are relatively large and expensive.

If the pulses arrive at rates that preclude their individual analysis, then current measurements are made. In this case, it is possible to employ electronics after the detector to amplify the analog current. Then, the final signal is given by Equation (16):

(16) $$E_{A}\;=\;E_{D}\;\times \;(\text{Amplification})$$

where E_A_ is the number of electrons after amplification. The electron arrival rate is a current, of course. Transimpedance amplifiers turn current values into voltages. For the case of pulse counting of photons, digital methods are used for computer recording of the photon arrival rates. For the analog current case, without or with amplification, analog-to-digital converters are usually used to obtain data in digital form for recording and manipulation by computers.

Whatever the means of spectral discrimination or photon detection and amplification, in or after the detector, both for digital photon counting and for analog current measurements, there usually results a digital signal related to the photon arrival rate at the detector.

### 2.10. No Optics

The preceding methodology dealt with lens coupling of the source photons to the sample and the coupling of either the transmitted source photons or generated fluorescent photons to the detector. Analytical microsystems without lens coupling are also possible. Their performance (calibration curves) can be computed using the equations already presented. Systems without intermediate optics can handle samples sizes over a wide range. Also, they are simpler than the case with lenses because there are fewer components to procure, align and hold in place. Without the constraint of the lens focal lengths, systems with no lenses can also be more compact. However, as will be seen in Section 3, the no-optics case has lower output signals for particular concentrations compared to systems with lenses.

### 2.11. Fiber Optics

The equations above provide the means to compute the calibration curves for microfluidic optical analytical systems using lens or no optics. As noted earlier, fiber optics can be employed to transport photons from the source to the sample and, thence, to a spectrally-sensitive component and detector. There are some notable advantages to using fiber optics with microfluidic systems. Because the external and core diameters of fibers can be comparable to the widths and depths of micro-channels, it is possible and relatively easy to integrate fiber optics into such fluidic platforms. This can be done by using ordinary fibers and putting them into the microfluidic platform, or by building optical channels, as well as fluidic channels, into a substrate. Either way, it is possible to closely couple an off-chip source to a fiber optic, which ends close to the fluid channel. Similarly, the space between the sample and a second fiber optic to take the fluorescent or unabsorbed photons to the filter before a detector, or to a spectrometer, can be small and geometrically efficient. It must be noted that fiber coupling is not attractive for single-use microfluidic platforms unless the fiber can also be disposable and easily (cheaply) connected to the unit containing the source, detector and electronics.

**Figure 5.**
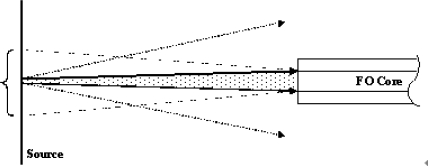
Diagram showing the part of the source (heavy vertical line) that is within the acceptance angle of a nearby fiber optic (indicated by the bracket), and the solid angle of light from one part of that region, which is intercepted by the core of a fiber (stippled).

The coupling of a source to a fiber optic is shown schematically in more detail in Figure 5. Two steps are needed to calculate the fraction of the emitted photons that enter the fiber. The first is to compute the fraction of the source area that is within the acceptance angle of the fiber. The next step is to calculate the fraction of the photons emitted from that area that fall on the core of the fiber optic. The result is Equation (17).

(17) $$P_{F}\;=\;P_{S}\times \frac{\Omega_{F}\cdot D^{2}}{A_{S}}\times \frac{{\pi R}_{F}^{2}}{\Omega_{S}\cdot D^{2}}\;=\;P_{S}\times \frac{\Omega_{F}\cdot {\pi R}_{F}^{2}}{A_{S}\cdot \Omega_{S}}$$

where P_F_ is number of photons entering the fiber optics, P_S_ is number of photons the source emitted, Ω_F_ is the acceptance solid angle of the fiber optics and the R_F_ is the core radius of fiber optics. D is the distance between light source and fiber. When Ω_F_·D^2^ is larger than source area A_S_, then Ω_F_·D^2^/A_S_ is equal to 1.

If the optical fiber acceptance specification is given as a numerical aperture (NA), Equation (18) permits calculation of half acceptance angle of fiber optic, θ_F_:

(18) $$\text{NA}=n\cdot \text{sin}\;(\theta_{F})$$

The refractive index n is 1 for air, 1.33 for water and 1.36 for ethanol. Equation (3) enables calculation of Ω_F_ from θ_F_. As in the case of a lens accepting radiation from a source, the emission pattern (solid angle) of the source enters the calculation. However, the very small fiber cores (on the order of 10 to 100 micrometers in diameter), rather than the lens diameter (on the order of 10 millimeters), are the acceptance areas.

It is interesting to note that, as the source-to-fiber distance D is increased, the area of the source viewed by the fiber increases as D^2^ while the area intercepted by the fiber core decreases as 1/D^2^. Hence, as long as the area of the source within the fiber acceptance angle is less than the overall source area, increasing D does not decrease the number of photons that get into the fiber. This presumes a source that emits uniformly over its area and over its solid angle.

There are two primary geometries for the coupling of light into and out of microchannels using fiber optics. They are orthogonal to the channel or co-axial with the channel. The transmission of incident photons for absorption measurements in the cross-the-channel case is relatively easy to compute using Equation (7). The beam coming from the fiber optic coupled to the source does not spread much when crossing a small channel.

The calculation of the number of unabsorbed photons is more complex in the co-axial case. Similarly, computation of the number of fluorescent photons generated, and the fraction captured by the fiber optic going to the detector, is not as simple in the coaxial case as in the lens coupling case. Calculation of both transmission and fluorescent signals for channels of varying lengths requires a single integration over the channel length. That is straightforward, but still more complex than the algebraic equations presented above. Coaxial couplings of microchannels and fiber optics are little used. Because of that fact, and because of their greater mathematical complexity, we do not present the integral equations for the coaxial case. However, results based on the use of these equations are given in the next section. It can be seen that coaxial coupling of microchannels and fiber optics leads to non-linear calibration curves at high concentrations and to very poor system efficiency.

The transmission efficiency of fiber optics is wavelength dependent. That efficiency may be gotten either from measurement or from the manufacturer's specifications in order to compute the fraction of the flux of photons from the source or sample that gets to the next part of the system.

### 2.12. Mixed Optics Systems

In the first part of this section, we dealt with systems having two lenses, one on each side of the sample in the micro-channel. Next, we dealt with the no-optics case. Then, we outlined the behavior of fiber optics that can be used in lieu of either of the lenses. It is possible to have optical micro-analytical systems that have mixed optics, with lenses, fiber optics or no optics either before or after the sample. For example, a lens might be used for an LED with a relatively broad emission solid angle to focus most of the source photons on the analytical fluid in a channel. Then, if a spectrometer with a fiber optic input is being used, it would couple the fluorescence from the sample into the spectrometer.

### 2.13. Overall Signal Calculation

The final expression, which relates the measured signal to the concentration or number of analyte molecules, can be gotten by successively linking the individual equations given above for the particular combination of components in any microfluidic analytical instrument. This is true for lens, no optics or fiber optics cases. For both fluorescence and absorption experiments, the signal depends linearly on the source strength. If the analyte fluid is optically thin to both incident and either fluorescent or transmitted radiation, then the signal also depends linearly on the number of molecules that are both irradiated by the source and viewed by the detector, whether it is an individual device behind a filter or built into a spectrometer.

The sensitivity of the signal to any of the geometrical and other parameters in the overall equation can be computed by taking the partial derivative of the signal strength with respect to the parameter of interest. In particular, the derivative of the signal with respect to the number of molecules is the slope of the calibration curve, that is, the instrumental responsivity, which is particularly important. A large derivative, that is, a high responsivity of the signal to the number of molecules, generally means that the precision of the analysis can be high, but the dynamic range will be relatively small. Conversely, a small slope and responsivity may make it possible for the instrument to give useful values over a broad range of molecular numbers (concentrations), but with less precision.

## 3. Computed Calibration Curves

The computational methodology just presented has been used to calculate the calibration curves for a wide variety of combinations of sources, optics, samples, detectors and geometries. While the methodology can be used for absorption analysis as well as for fluorescence situations, we concentrate on the fluorescence approach. Most of the published papers on microfluidic optical analysis use fluorescence rather than absorption. And, the measurements we are planning to test the new computational methodology are based on fluorescence and not absorption. Besides, the computation of the source absorption in the process of estimating the fluorescence intensity is essentially the same as the calculation of signals for absorption experiments.

The results of our calculations of fluorescence calibration curves presented in this section are based on particular optical components and their specifications. The specific components for which we have done calculations and are doing experiments will be cited in detail in experimental papers.

**Figure 6.**
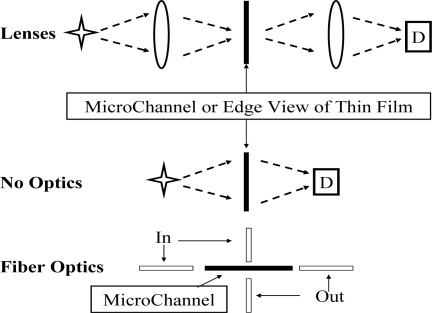
The three cases for which calculated calibration curves are presented. The first is lens coupling to and from either microchannels or thin films. The second case has the same types of sample holders, but without optics. The last case deals with fiber optics coupling to a micro-channel, either within (co-axial) the channel or else orthogonal (cross) the channel.

Since the optical coupling and geometry are major variables in both the design and performance of microfluidic optical analytical systems, we employed three different cases, which are presented in Figure 6. The computational results are based on these three geometrical cases, and on using three different light sources, three different optics, two different samples and two detectors. The detector outputs for six concentrations were computed for each sample and combination of components and geometry. Hence, the information presented here is the result of over 600 individual calculations of detector output for specific combinations of components, geometries and concentrations all done using an EXCEL spreadsheet. Over two thousand computations were done with the spreadsheet in order to examine alternative geometries. This testifies to the facility with which calibration curves for optical micro-analysis can be computed using our methodology.

**Figure 7.**
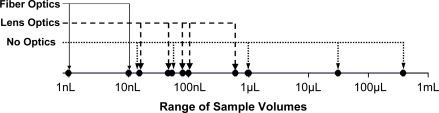
The volumes of samples for which the results in this section were obtained. 1 nL is a cube 100 micrometers on a side. 1 mL is 1 centimeter cubed. Fiber optics are small and interrogate only small volumes. Systems with no optics can probe a wide range of volumes, including relatively large samples.

The volumes of the samples, which are analyzed for these three types of optics, are plotted in Figure 7. It is noteworthy that our methodology has handled samples that range in volume from 1 nL to about 1 mL. Computation of calibration curves for smaller or larger samples is also possible with this methodology. We used fluorescein for the illustrative calculations because it has been widely employed in experiments with microfluidic analytical systems [11–17].

**Figure 8.**
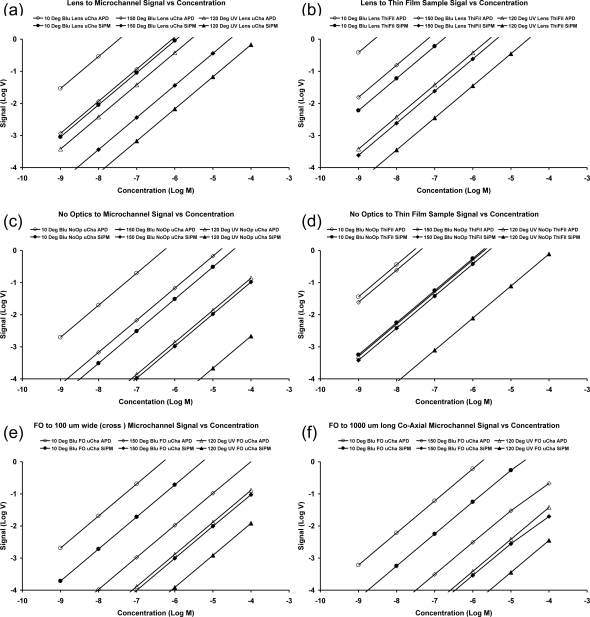
Computed calibration curves as a function of the molar concentration of fluorescein for several geometries, sources, optics and detectors: (a) lens (1.5 cm focal length and diameter) coupling to a 100 μm square microchannel, (b) lens (1.5 cm focal length and diameter) coupling orthogonal to a 100 μm thin film, (c) light from sources to 100 μm square microchannels and fluorescence to detectors without intervening optics and (d) light from sources to 100 μm thin films and fluorescence to detectors without intervening optics, (e) fiber optic (100 μm diameter ) coupled orthogonal (cross) to a 100 μm square microchannel, (f) fiber optic (100 μm diameter) coupled within (co-axial) a 1,000 μm length of a 100 μm square channel. The sources are blue LEDs with either 10 or 150 degree full emission angles or a UV LED with a 120 degree full emission angle. A filter was employed and the transmission loss through the filter was calculated, as described in Section 2.7. The detectors are either an amplified photo diode (AmPD) or a Silicon photomultiplier (SiPM). The straight lines are drawn through the computed points in these graphs.

**Figure 9.**
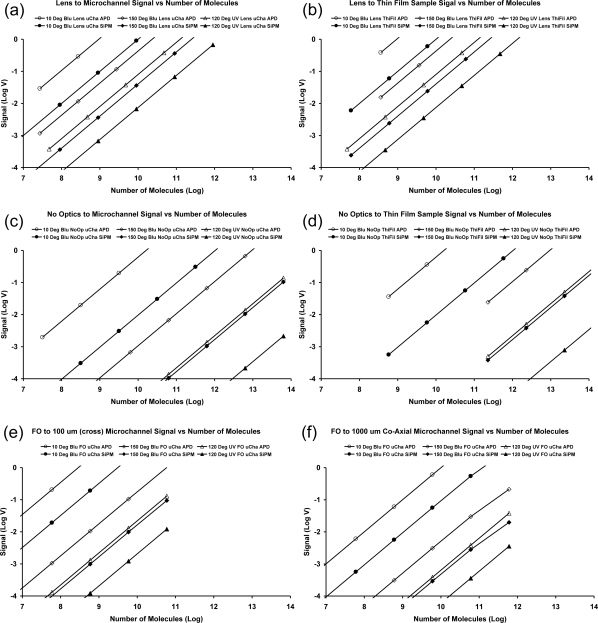
Computed calibration curves as a function of the number of fluorescein molecules for several geometries, sources, optics and detectors. This figure is made by converting the concentration into number of molecules in different volumes shown in Figure 7. Because of the sample volumes are different in various geometrical arrangements, the number of molecules is different at any concentration.

Calibration curves were computed as a function of both concentration (molarity) and the numbers of molecules accessible in the analysis. Concentrations are commonly desired, but the numbers of olecules are useful for comparing the efficiencies of optical analytical instruments. The calibration curves for the three geometrical cases of Figure 6, and many component variations, are presented in Figures 8 and 9. The calculated curves have the same slopes because all parts of the systems are linear. The use of log-log scales is necessary because of the very wide ranges of concentrations and output signals. These graphs clearly show the absolute and relative performance of the various components and geometries. Vertical lines at specific concentrations would show that the signals from the detectors can vary by over three orders of magnitude for a particular concentration. Horizontal lines can be used to bracket the detector outputs ranging from the noise level to the saturation signal. The minimum detectable limit and the dynamic range vary greatly depending on the optical components and their arrangements.

The computed signals for specific concentrations or number of molecules vary more than 10^3^ for the different components and geometries. It is clear that the case for the analyte in microchannels and coaxial light transmission gives relatively poor performance. Conversely, having the sample in a thin film with both the incident excitation light and fluorescence at 90 degrees to the film provides much greater signals than the other cases.

We emphasize the fact that our methodology does not provide absolute computed noise limits for calibration curves nor absolute computed maximum available signals. However, obtaining these characteristics is not a practical problem. The intersections of the calibration curves, which are computed, with the minimum detector signal gives the minimum detectable concentration (MDL). The MDL will be improved if the detector has a lower noise floor. Similarly, the intersections of the calibrations curves with the maximum detector outputs give the highest concentration that can be assayed, and hence, the dynamic range of the system. The MDL and dynamic range for the various calibration curves were determined using the published characteristics for the two detectors. The silicon photomultiplier (SPMMicro1000X01A1from SensL) has a noise floor of 1 mV and a maximum signal of 500 mV. The amplified photodiode (Model ODA-6WB-500M from OptoDiode) has the same noise floor and a maximum output of 5 V when supplied with voltages equal to ±5 V. These values were employed in determining the MDL and dynamic ranges for the 36 combinations in Figures 8 and 9. The results are presented in Table 1.

## 4. Discussion of the Results

The tabulation of MDL values and dynamic ranges makes easier the evaluation of the results of the computations compared to use of the log-log graphs already presented. Considering the MDL for the various cases, the values range from 3 pico-molar to 46 micro-molar, a variation of over 10^7^. The facility with which these calculations were done and the wide variation in results illustrates the value of our methodology for micro-analytical system design and comparison. The narrower emission angle LED light source is more efficient for delivering the photons to excite the fluorescence emission compared to the same LED with little collimation. Lens coupling shows better incident photon transmission from an LED light source to the sample along with better fluorescence photon delivery from the sample to the detector. However, it must be re-emphasized that we put the source on one side of the assumed-transparent substrate containing the channel or thin film and the detector on the other side. This is not a practical geometry because light from the source would enter the detector. Placing the source and detector on the same side of the substrate would essentially remove this problem, but decrease the geometric coupling slightly.

The MDL values in Table 1 show that the thin film sample geometry is substantially better for all combinations of sources, samples and detectors. This is because the useful part of the thin film sample contains more molecules due to having a bigger volume. It is a good trade-off to use larger volume of sample (that is micro-liters, rather than nano-liters) in order to reach lower MDL. One ordinary drop contains about 50 μL.

## 5. Conclusions

Optical microfluidic systems are widely employed in micro-analytical research and industry [18]. Examination of the alternatives we considered leads to an appreciation of the large number of possible optical micro-analytical systems. We discussed multiple photon sources; lenses, fiber and no optics for photon transport; fluorescence and absorption techniques for probing samples in micro-channels and thin films; filters and spectrometers for spectral discrimination; and various detectors with anallary electronics. There are many specific choices in each of these categories. Hence, there are hundreds of specific systems. All of these can be analyzed and compared quantitatively using our methodology.

The largest photon loss in an optical analytical system occurs when there is no efficient way to collect fluorescence photons from the sample to the detector. Whether the system has lenses or fiber optics, there is a numerical aperture (NA) or acceptance angle for each optic. It determines the efficiency for gathering the fluorescence photons from the sample that are emitted into 4π steradians. Light gathering efficiency is a key factor in designing microfluidic analysis systems that can provide lower MDLs. Use of ellipsoidal or other mirrors to gather fluorescent photons was not computed for this paper. However, this methodology can be confidently employed for those cases.

Comparisons of computed and measured calibration curves, both with the same units, should prove especially useful. We note the central importance of the absorption coefficients in both fluorescent and absorption methods and of the fluorescent yields in fluorescent measurements. It may be possible to obtain relative or absolute experimental estimates of these parameters for particular combinations of analyte molecules and wavelengths using our methodology. This requires that all the geometrical and other parameters are known, or can be independently measured, with sufficient accuracy. In particular, the absolute source strength, and the quantitative performance of the detector and subsequent electronics, must all be known. Determination of absorption and fluorescence parameters is challenging. However, if such values are not available, comparison of the computed and measured signal strengths could give estimates for the absorption coefficients and fluorescent yields. It remains to be seen if this approach has usefully small errors. Determining that would be one of the motivations for performing an experimental assessment of the methodology.

## Figures and Tables

**Table 1.** The minimum detection limits (MDL) in nM and dynamic ranges (factors above the MDL in parentheses) for the calibration curves for the three sources, three optics options, three sample geometries, and two detectors.

**Optics**	**Sample**	**Detectors**	**Light Sources**		
			**10 Deg Blue LED**	**150 Deg Blue LED**	**120 Deg UV LED**
Lenses	100 μm Wide Microchannel	SiPM	1.11 (473)	27.58 (11,700)	148.53 (63,200)
Lenses	100 μm Wide Microchannel	Amplified Photodiode (AmPD)	0.03 (173)	0.86 (4,300)	3.98 (19,900)
Lenses	100 μm Thin Film	SiPM	0.17 (71)	4.14 (1,760)	28.37 (12,100)
Lenses	100 μm Thin Film	Amplified Photodiode (AmPD)	0.003 (13)	0.06 (320)	2.65 (526)
None	100 μm Wide Microchannel	SiPM	32.67 (139,020)	958.52 (408,000)	46622.98 (20,000,000)
None	100 μm Wide Microchannel	Amplified Photodiode (AmPD)	0.51 (2,550)	14.93 (75,000)	726.11 (144,000)
None	100 μm Thin Film	SiPM	1.78 (760)	2.64 (1,100)	128.06 (55,000)
None	100 μm Thin Film	Amplified Photodiode (AmPD)	0.03 (140)	0.04 (200)	1.99 (400)
Fiber Optics	100 μm Microchannel (cross)	SiPM	5.25 (2,630)	1000 (523,800)	8128.3 (4,065,670)
Fiber Optics	100 μm Microchannel (cross)	Amplified Photodiode (AmPD)	0.48 (2,510)	95.5 (490,000)	758.58 (3,900,000)
Fiber Optics	1,000 μm Microchannel (co-axial)	SiPM	17.78 (10,450)	3801.89 (2,235,000)	28183.83 (14,100,000)
Fiber Optics	1,000 μm Microchannel (co-axial)	Amplified Photodiode (AmPD)	1.55 (9,770)	346.73 (2,190,000)	2630.27 (13,180,000)

**Figure A-1.**
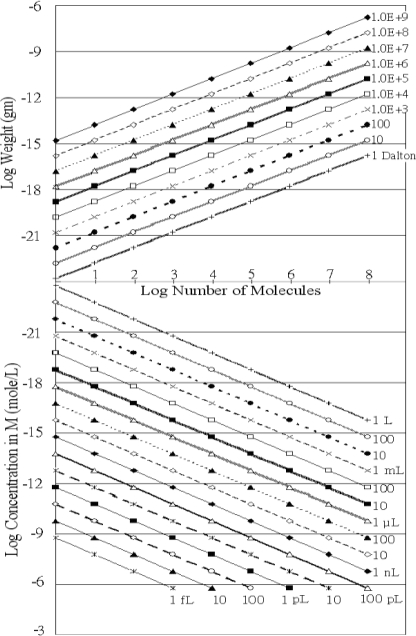
Two sets of graphs relating the number of analyte molecules to the sample volume and concentration (bottom) and to the molecular and total weights (top).

## Appendix: Parametric Relationships

There are five parameters that are relevant to the analyte in a microfluidic platform. They are (1) the number of molecules and (2) the analyte volume that are within the acceptance geometry of the optical system. Together, these determine (3) the concentration of the molecules of interest in the sample. If (4) the molecular weight is known, then it and the number of molecules give (5) the mass of the molecules within the viewed part of the sample. Because of the relationships between these quantities, they can be shown together graphically, as indicated in Figure A-1.

The number of molecules is most important and, hence, is common to the two sets of graphs. That number and the volume give the concentration (molarity) in the bottom of the figure. The number and molecular weight give the absolute weight of the analyte molecules in the top of the figure. The overall weight can also be related to the volume and density of a dry, solid particle of the molecules of interest that might be captured and dissolved for microfluidic analysis.
